# An optical interferometric technique for assessing ozone induced damage and recovery under cumulative exposures for a Japanese rice cultivar

**DOI:** 10.1186/2193-1801-3-89

**Published:** 2014-02-14

**Authors:** Bodhipaksha Lalith Sanjaya Thilakarathne, Uma Maheswari Rajagopalan, Hirofumi Kadono, Tetsushi Yonekura

**Affiliations:** Graduate School of Science and Engineering, Saitama University, Saitama, Japan; Laboratory for Integrative Neural Systems, RIKEN Brain Science Institute, 2-1 Hirosawa, Wako City, Saitama 351-0198 Japan; Center for Environmental Science in Saitama, Kazo, Saitama Japan

**Keywords:** Crop, Rice (*Oriza sativa* L.), Growth, Plant growth measurement, Atmospheric pollution, Optical interferometric technique, Ozone, Chlorophyll fluorescence, Environmental effects, Instantaneous growth, Yield

## Abstract

Exposure to ozone (O_3_) causes reduction both in the growth and yield of rice (*Oriza sativa* L.). Commonly used Chlorophyll fluorescent measurements are not sensitive enough for short term exposure of O_3_ aiming an immediate assessments. Such a conventional method typically needs exposure over a few days to detect the influence. As an alternative method, we proposed a novel non-invasive, robust, real-time, optical Statistical Interferometric Technique (SIT) to measure growth at an accuracy of 0.1 nm with a commonly consumed Japanese rice cultivar, Koshihikari. In the present study, we have conducted a repetitive O_3_ exposure experiment for three days under three different concentrations of 0 nl l^-1^ (control), 120 nl l^-1^, and 240 nl l^-1^, to investigate the damage and recovery strengths. As a measure to assess the effect and recovery from three consecutive day exposures of O_3_, we measured the elongation rate (nm mm^-1^ sec^-1^) every 5.5 sec for 7 hours, and it revealed nanometric elongation rate fluctuations or Nanometric Intrinsic Fluctuations (NIF). Comparing the standard deviation (SD) of normalized nanometric intrinsic fluctuations (NNIF), which was normalized by that before the exposure, we found that drastic reductions under both 120 nl l^-1^ and 240 nl l^-1^ O_3_ concentrations. Reduction percentages were large under high O_3_ concentration of 240 nl l^-1^ indicating the possibility of irreversible effect. However exposure to 120 nl l^-1^ of O_3_ showed recovery on the 2^nd^ and 3^rd^ days. While SIT did reveal immediate effect based on an observation for a few hours, the visible foliar effect could be observed only after a week. Hence, the technique could provide a way for fast assessment of effect and recovery due to cumulative exposure of O_3_ and hence the tolerance as well as the vitality of plant.

**Electronic supplementary material:**

The online version of this article (doi: 10.1186/2193-1801-3-89) contains supplementary material, which is available to authorized users.

## Introduction

Tropospheric ozone (O_3_) is an air pollutant generated by the photo oxidation of precursor gases, such as NOx and volatile organic compound (VOC) (Ainsworth 2008; Ashmore 2005; Darrall 1989). Further, O_3_ concentrations exceeding 80 nl l^-1^ occur regularly, even in rural areas of India, China, and Japan (Beig et al. 2007; Wang et al. 2007; Kobayashi et al. 1995; Kobayashi and Okada 1995). According to projections, O_3_ pollution may cause rice yield losses of up to 16% with no change in agricultural practices (Ainsworth 2008), which would put food security in Asia at a substantial risk.

It is known that O_3_ causes biochemical and physiological changes leading to the inhibition of photosynthesis and a consequent decrease in plant growth. O_3_ enters the leaf through stomata, the pores for photosynthetic gas exchange (Fiscus et al. 2005; Roelfsema and Hedrich 2005). Adverse effects on plant photosynthesis were identified as a major factor limiting growth and yields of crop under high O_3_ concentrations. Another mechanism, which affects plants, is through the oxidative damage. The breakdown of O_3_ in the apoplast is thought to lead to the formation of reactive oxygen species (ROS), such as superoxide, hydrogen peroxide, and hydroxyl radicals. Such an O_3_ -induced oxidative burst results in tissue damage that produces visible leaf damage leading to ROS-induced cell death process (Fiscus et al. 2005; Rao and Davis 2001; Frei et al. 2008; Pell et al. 1997; Black et al. 2007).

Studies found that the acute O_3_ concentration of 560 nmol mol^-1^ h could result in visible injury. This was obtained from accumulated exposure over duration of 7 hours for eight consecutive days (Karlsson et al. 2004). Most of the researches carried out so far have been done with main focus on investigating the damages in biochemical, cellular, physiological, and morphological level of plants under chronic O_3_ exposure conditions for a relatively long period of a few weeks or few months (Cosgrove 2000; Zheng et al. 2000; Guidi et al. 2007; Gill and Tuteja 2010).

As a way, to assess the effect of O_3_ on plant growth fast, we propose the use of Statistical Interferometric Technique (SIT), a non-invasive and non-contact optical interferometric method (Kadono et al. 2001; Kadono and Toyooka 1991). For measuring growth at higher resolutions, attempts were made to use optical interferometer (Fox and Puffer 1976; Fox and Puffer 1977; Briers, 1977; Jiang and Staude 1989). An optical interferometer involves the interference of reflected light from an optically flat reference mirror and that from a plant. Usage of an interferometer was limited because of two main factors. One is the complexity of the implementation and the other being, the optical property of the plant itself. Plant surface is highly scattering from both stationary and moving organelles on the surface and those within the leaf. The unwanted scattered light leads to the formation of a random pattern, called speckles (Dainty 1984), obstructing the interferometer to achieve the expected accuracy. In SIT, the totally random wave or speckle was used as reference instead of a plane wave generated from an optically flat mirror used in a conventional interferometer (Hariharan 1985).

We demonstrated SIT to be capable of measuring plant growth at a spatial scale of nanometer (nm) and at a temporal scale of second (sec) under various environmental changes such as under O_3_ stress (Rathnayake et al. 2007) of Japanese red pine roots (*Pinus densiflora* Seibold & Zucc.) and infection of roots by two ectomycorrhizal fungi (Rathnayake et al. 2008). Kobayashi and Kadono (2010) reported the effect of illumination conditions on the leaf-growth of rice crops by using SIT.

In our recent investigation on the measurement of leaf-elongation of two different rice crops, Koshihikari and Fusaotome rice cultivars, it was revealed that the elongation rate contains random fluctuations at the nanometric spatial scale, nanometric intrinsic fluctuations (NIF). From characterizing these NIF by standard deviation, we found that NIF was influenced by environment such as the presence of O_3_. The NIF discovered differed between the cultivars and were also found to be present in different plants (Thilakarathne BLS, Rajagopalan UM, Kadono H, Yonekura T, High speed and high precision optical interferometric technique to investigate instantaneous growth related changes of plant leaves, submitted).

In this study, we focused on NIF and its dependence on repetitive cumulative O_3_ exposures of 7 hours for three consecutive days. O_3_ concentrations of 0 nl l^-1^, 120 nl l^-1^, and 240 nl l^-1^ were used. A Japanese rice cultivar, Koshihikari was chosen for its high sensitivity to O_3_ (Yonekura 2011; Thilakarathne BLS, Rajagopalan UM, Kadono H, Yonekura T, High speed and high precision optical interferometric technique to investigate instantaneous growth related changes of plant leaves, submitted).

## Results and discussion

### Leaf elongation rate under repetitive ozone exposures

**Figure 1:**
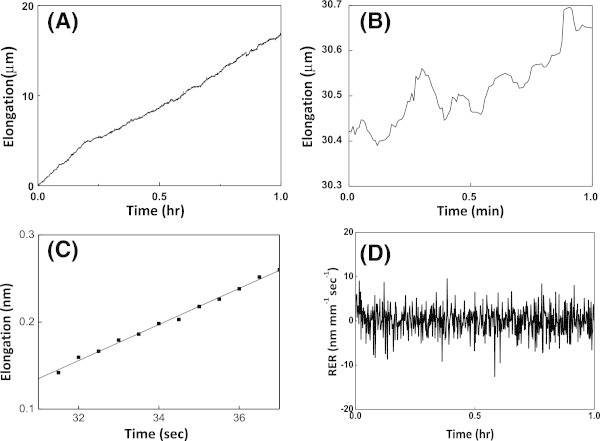
**Elongation of the leaf were continuously measured over 7 hours and shown at different time scales.** Elongation of the leaf were continuously measured over one hour and shown at different time scales **(A-C)**, and relative elongation rate for the leaf **(D)**. The results were shown for an hour **(A)**, a minute **(B)**, and a few seconds **(C)**. In **(C)**, the straight line corresponded to a linear regression done to obtain the elongation rate. Elongation rate variation over an hour showed random fluctuations at nm spatial scales in conjunction with the monotonous macroscopic growth **(D)**. Noise level of the system was measured with a metal plate as a sample, and the standard deviation of the fluctuations obtained with the metal plate was less than 0.1 nm mm^-1^ sec^-1^.

The elongation data of Koshihikari leaf obtained under different time periods of minute to seconds under control O_3_ concentration of 0 nl l^-1^ are shown (Figure 1(A)-(C)). Over an hour, the elongation was in the order of tens of μm and almost monotonically and slowly varying (Figure 1(A)). When confined to within less than a minute, random elongation fluctuations were observed in growth of a few hundred nanomters (Figure 1(B)). Under a few seconds as shown here for 6 sec, the elongation was almost linear and can be fitted to a line by linear regression (Figure 1(C)) to obtain the elongation rate. This elongation rate was normalized by the beam distance to calculate the relative elongation rate (RER).

Figure 1(D) shows the relative elongation rate variation over 1 hour. The elongation rate was rapidly varying appearing as random noise fluctuations. It was found that the standard deviation (SD) of the fluctuations for the metal plate, which determine the noise level of the measurement system, was more than twenty times smaller than that for the leaf.

**Figure 2:**
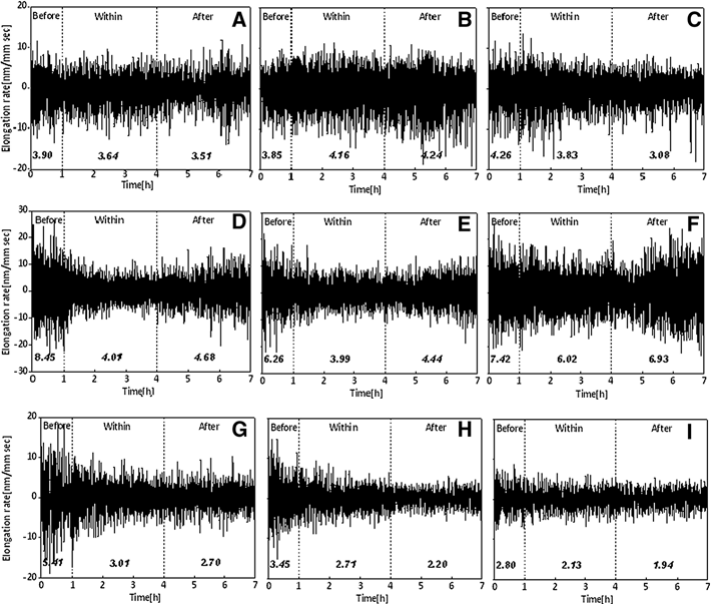
**Results of nanometric intrinsic fluctuations (NIF).** Results of nanometric intrinsic fluctuations (NIF) showing the effect of O_3_ under expeosure conditions of CF air (top row, **(** **A-C** **)**), 120 nl l^-1^ (middle row, **(** **D-F** **)**), and 240 nl l^-1^ (bottom row, **(** **G-I** **)**) of O_3_ obtained on day 1 (leftmost column, **(A, D, G)**), day 2 (middle column, **(B, E, H)**) and day 3 (rightmost column, **(** **C, F, I** **)**). In figure, the durations ‘Before’, ‘Within’, and ‘After’ correspond to CF air before O3 exposure, within the exposure, and CF air after the exposure, respectively, and are shown by separating with broken lines.

Figure 2 shows the elongation rate fluctuations observed under three different O_3_ exposure conditions 0 nl l^-1^ (top row), 120 nl l^-1^ (middle row), and 240 nl l^-1^ (bottom row) with each row containing data obtained on day 1 (left most), day 2 (middle), and day 3 (right most). The periods ‘Before’, ‘Within’, and ‘After’ correspond to charcoal filtered (CF) air before O_3_ exposure, within the exposure, and CF air after the exposure, respectively, and are shown by separating with broken lines. The fluctuation amplitudes decrease with increase in concentration as well as due to cumulative exposures.

In order to characterize these changes with exposure and repetition, the SD of these fluctuations was used as a norm and was referred as nanometric intrinsic fluctuations (NIF). Moreover, in order to reduce the common variations during a single session for a sample as well between different samples, normalization was done. The standard deviation of the fluctuations calculated under the initial one hour of CF air, *SD*_*before*_, was used as the normalizing factor. *SD*_*exposure*_ and *SD*_*after*_, standard deviations of the fluctuations under three hours O_3_ exposure, and under the last three hours of CF air exposure were normalized by *SD*_*before*_. The normalized fluctuations were called as normalized nanometric intrinsic fluctuations (NNIF).

### Normalized nanometric fluctuations for characterizing ozone effect

**Figure 3:**
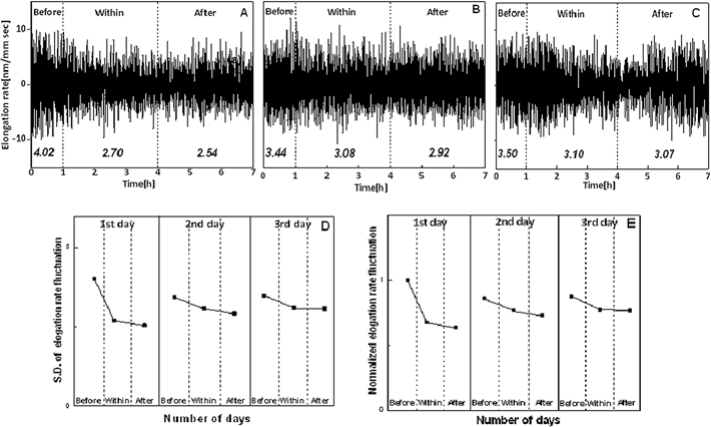
**Normalization procedure.** Normalization process during each time period (before, within, and after) in three days for Koshihikari rice sample. Elongation rate variations shown as a function of time over three days **(A-C)**. Variation of standard deviation as a function of time obtained during different time intervals **(D)**. Variation of normalized standard deviations of elongation rate **(E)**. Here standard deviation obtained at ‘Before’ on the first day was taken as the normalization factor. In all figures, **(A)**-**(C)**, the durations ‘Before’, ‘Within’ and ‘After’ are shown by separating with broken lines.

NNIFs were calculated for all six samples under each O_3_ concentrations of 0 nl l^-1^, 120 nl l^-1^, and 240 nl l^-1^ and then averaged to give averaged NNIF. Figure 3 showed the normalization procedure. The elongation rate fluctuations obtained from a leaf for three days are shown in Figure 3(A-C). The SDs during each time period of CF air before, 120 nl l^-1^ O_3_ exposure, and CF air after were calculated and shown in Figure 3(D). Figure 3(E) showed the normalized standard deviations or NNIFs.

**Figure 4:**
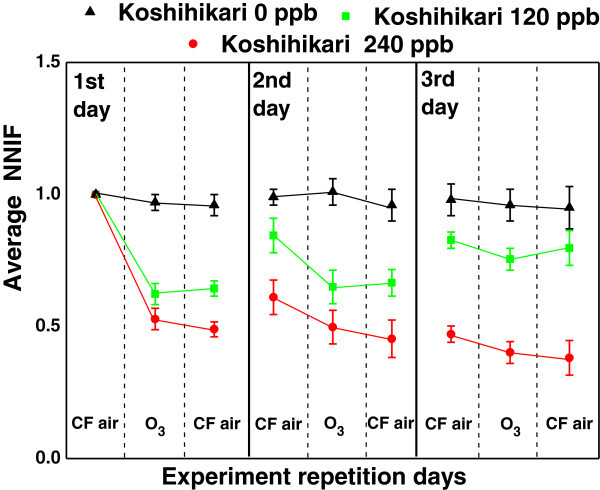
**Results of averaged standard deviation of NNIF.** Results of averaged standard deviation of NNIF for Koshihikari rice cultivar showing the effect of O_3_ under three experimental conditions of 0 nl l^-1^, 120 nl l^-1^, and 240 nl l^-1^ O_3_ exposures for three consecutive days. The error bars correspond to the SEs of the mean. Shown are means ± SE. The durations ‘Before’, ‘Within’ and ‘After’ are shown by separating with broken lines. For each treatment (CF, 120 nl l^-1^, and 240 nl l^-1^) six rice samples from Koshihikari cultivar was used. All together 18 samples were investigated.

Figure 4 showed the variation of NNIF as a function of repetition day under three different O_3_ conditions of 0 nl l^-1^, 120 nl l^-1^, and 240 nl l^-1^. It can be clearly seen that, for 0 nl l^-1^, there was almost no variation across the measurement duration and also across days. This indicated the measurement was relatively stable during the total period of three days.

### Results of repetitive ozone exposure for three days

Both under 120 nl l^-1^ and 240 nl l^-1^, on the first day, a clear decrease in average NNIF could be seen within exposure period. On the first day of the exposure of 120 nl l^-1^, measurement showed that there was no recovery to the pre-exposure level within the first day. However, on day 2, an increase in average NNIF could be seen at the start of the experiment, indicating a possible recovery of the plant system from O_3_ stress on the first day. Again, with three hour exposure 120 nl l^-1^ O_3_ on second day, there was a decrease in average NNIF but the decrease was getting smaller possibly due to acclimation of the plant to the O_3_ induced stress. On third day, there was actually increase again indicating the recovery with a reduced extent.

For the case of 240 nl l^-1^, the decrease in fluctuation amplitude was large, and with consecutive day exposures there was little difference in the decreases between the days. From Figure 4, we can see that there was a definite change in the amplitudes with repetitive exposure. We can also see some adaptation and the adaptation behavior depends on the concentration of O_3_. While for 120 nl l^-1^, the plant’s vitality is trying to get close to the pre-exposure level, the O_3_ effect becomes large enough that there was almost no change in the amplitudes with consecutive exposures for 240 nl l^-1^.

Statistical analysis showed a significant difference (p < 0.01) between control and 120 nl l^-1^ exposure, and control and after 120 nl l^-1^ exposure. This was true for 240 nl l^-1^ too. Table 1 shows the decrease in amplitudes in percentages for three different conditions obtained under 0 nl l^-1^, 120 nl l^-1^, and 240 nl l^-1^.

**Table 1:** **Reduction percentages in NNIF**

Repetition day	% decrease in amplitude for 0 nl l^-1^			% decrease in amplitude for 120 nl l^-1^			% decrease in amplitude for 240 nl l^-1^		
	Before	Within	After	Before	Within	After	Before	Within	After
Day 1		97	96	-	62	64	-	52	49
Day 2	99	101	96	84	65	67	61	50	45
Day 3	98	96	95	83	75	80	47	40	38

Decrease in NNIF with repetitive three day exposures under O_3_ concentration of 0 nl l^-1^, 120 nl l^-1^, and 240 nl l^-1^. The decreases were those obtained during three time periods of before, within and after of O_3_ exposure.

### NNIF and repetitive ozone exposure

Due to the potential that SIT can monitor the changes not only before and after but also during the exposure of O_3_ making it a promising technique for monitoring continual changes in comparison to other techniques, we conducted an investigation with a continual exposure. In this study, leaf of Koshihikari cultivar was chosen for its relatively high sensitivity to repetitive exposure of O_3_ for three consecutive days. We found that there was no decrease in the normalized factor NNIF under control, i.e., under an exposure of concentration 0 nl l^-1^ on all three days. This suggests that our measurements were done in an almost robust condition during the total experimental period in relatively stable system and were not affected by external fluctuations like temperature and humidity.

Under O_3_ exposure of concentration 120 nl l^-1^, there was respective immediate decrease in NNIF by 38% on the first day with no recovery back to pre-exposure state within the day. However, on the start of the second day, there was a recovery by 20% which started to show immediate decrease with exposure reaching a total decrease to 67% on the second day. On the third day, for 120 nl l^-1^ recovery could be seen by 16% that remained almost constant throughout third day. The overall decrease compared to pre-exposure was less than 25%. This indicated the recovery of the intrinsic adaptation of the system to continual exposure, although the recovery could never reach the pre-exposure state.

In contrast, under 240 nl l^-1^, the decrease on the first day itself was large by 51% and there was no significant change in decrease of NNIF between second and third days. While at the end of the second day the fluctuations decreased to 45% and on the end of the third day the fluctuations decreased to 38%. Therefore, the fluctuations became small that the deviations were much below the pre-exposure value at the first day and could never approach the pre-exposure level of NNIF. In actual case for 240 nl l^-1^, foliar damage could be seen only after a week of the three consecutive day exposures. The current investigation demonstrates the potential of SIT in immediate assessment of O_3_ exposure damage.

### Origin of NIF

The observation using SIT is basically a macroscopic one since the probing region is the order of mm. The subnanometer accuracy of SIT makes it possible to monitor molecular or cell expansion changes as an integration of expansion changes over many cells. We hypothesize that the nanometric fluctuations are from possibly physiological changes accompanying plant growth. For growth of plant cells to happen, the cells must physically expand their restraining walls. At the same time the wall must preserve its mechanical integrity in the presence of high turgor pressures. Spatial expansion may vary depending on the part of the plant and also on the various cell specific patterns of enlargement and divisions. Existence of a variety of cell growth patterns and cell-specific enlargement leads to a variety of cell shapes. The spatial patterning is regulated by a variety of enzymatic processes that lead to modifications in the cell wall (Cosgrove 2000; Pantin et al. 2011).

#### Sensitivity of NIF and O_3_ concentration

Previous studies do indicate that the exposure to O_3_ results in the reduction of both live regulating metabolic and hydraulic processes (Roelfsema and Hedrich 2005; Rao and Davis 2001; Black et al. 2007;Ruts et al. 2012; Wilkinson and Davies 2010; Kangasjärvi et al. 2005). As the nanometric fluctuations based on the results are hypothesized to be related to life regulating processes, we expected a decrease in NIF with increasing O_3_ accumulation. In SIT, the measured NIF decreased immediately for large O_3_ concentration.

**Figure 5:**
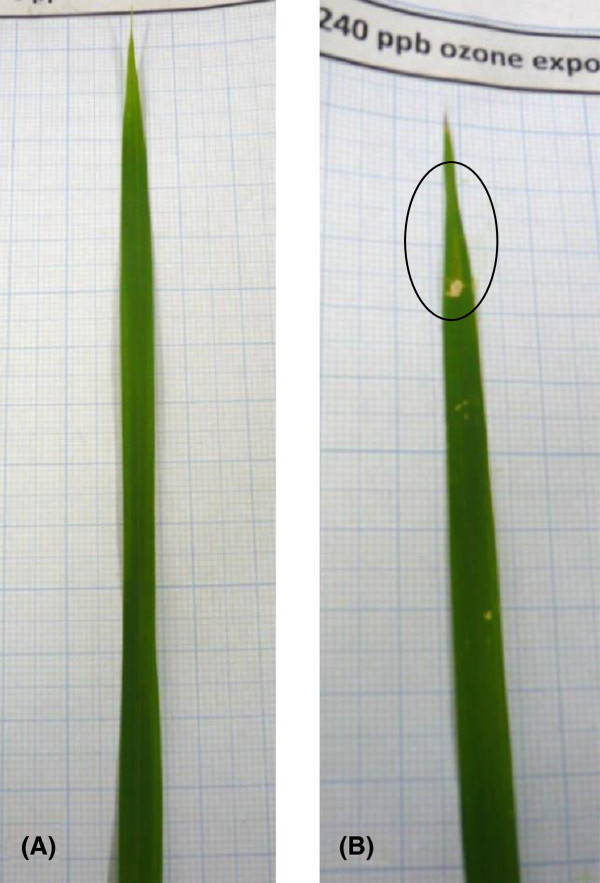
**Foliar damage observed after a week.** Foliar damage observed after a week for three days with O_3_ exposure of 0 nl l^-1^ **(A)** and 240 nl l^-1^ **(B)**. The yellowish spot seen on illuminated spots of the leaf and on the tip resulted from the consecutive exposure of O_3_ for three consecutive days. Black color circle showed foliar damage area. The damage appeared after a week from the start of the exposure. The damage was visible only under 240 nl l^-1^ and not under 120 nl l^-1^ although under both levels the damage was irreversible. The illumination spots were identified based on the flour remaining on the leaf after finishing the exposure experiments.

In the current investigation, it was found that with repetitive exposure for three days of O_3_ under relatively small dosage of 120 nl l^-1^, it was possible to see the recovery while under a relatively large dosage of 240 nl l^-1^ for three days, the decrease in NIF was large and it was possible to observe some recovery on the second day that became almost null on third day. The irreversible damage in the leaf could be seen as foliar damage (Figure 5) only after a week. This agrees again with the earlier agreement of the results in reduction with gas exchange measures under 240 nl l^-1^ (Thilakarathne BLS, Rajagopalan UM, Kadono H, Yonekura T, High speed and high precision optical interferometric technique to investigate instantaneous growth related changes of plant leaves, submitted).

The physiological basis behind the origin of the fluctuations and the influence of the environment needs a further detailed investigation. The current study only focused on reporting the presence of such fluctuations and their sensitivities.

## Conclusions

In this paper, we applied SIT for measuring the RER of a relatively low O_3_ tolerant Japanese cultivar, Koshihikari, to investigate its dynamic response to three hour O_3_ exposure for three consecutive days at O_3_ concentrations of 0 nl l^-1^, 120 nl l^-1^, and 240 nl l^-1^. RER measurement at a temporal resolution of 0.5 sec and subnanometor accuracy revealed nanometric intrinsic fluctuations, NIF. To compare NIF across three days as well between different O_3_ exposure concentrations, SD was used as a norm and normalized with those obtained on 1^st^ day before the exposure and averaged across six samples to give NNIF. NNIF was found to vary with the exposure concentrations of O_3_, and also NNIF to repetitive exposures revealed the dynamic response of possible recovery of the leaf under 120 nl l^-1^ O_3_ exposures. On the other hand, for an exposure of O_3_ concentration, 240 nl l^-1^ was found to produce a large damage on the first day of the exposure. Although the following days’ exposures indicated the evidence for the existence of recovery mechanisms, there was continual damage finally resulting in the appearance of foliar damage after a week. The possibility of monitoring ongoing response of the plant to environmental changes makes SIT an attractive tool. Conventional techniques such as gas exchange measures basically obtain data before and after change in environment and it is not possible to measure in a dynamic way.

## Methods

### Experimental system of SIT

**Figure 6:**
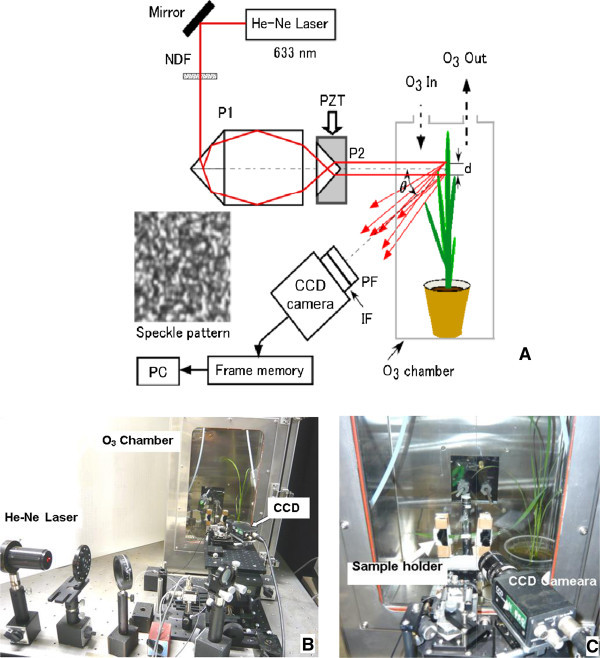
**A schematic of the experimental system of SIT.** A schematic of the experimental system of SIT **(A)** used for measuring plant growth placed in an O_3_ chamber with a photo of the real system on an optical bench **(B)**. Third or fourth leaf was stably fixed using a sample holder **(C)** with precaution taken to avoid any damage to the leaf. The leaf length was about 15 to 20 cm. The probing points on the leaf were set to be around 4 cm from the apex of the leaf. The distance *d* between the points of illumination on the leaf was set to be 3 mm, and *d* can be adjusted by moving the prism P2 along the optical axis. The CCD camera was placed at a distance of 83 mm from the probing area, and the speckle interference patterns **(A)** were continuously recorded. Here, the notations indicate, NDF: neutral density filter, PZT: piezoelectric transducer, P1 and P2: prisms to generate two parallel probing beams, *θ*: angle between the probing and observing directions.

Figure 6(A) shows a schematic of the statistical interferometer assembled on an optical bench (Meiritsu seiki, Japan) used to measure plant growth. A photograph of the optical system is given in Figure 6(B). The light emerging from a He-Ne laser of wavelength of 633 nm (GLG 5400, NEC corporation, Japan) was first passed through a neutral density filter (NDF) (F71N-2, Suruga Seiki, Japan) to adjust the light intensity. The light was further divided into two beams by a specially designed prism P1 (Suruga Seiki, Japan). Two beams normally illuminated two points on the surface of a growing leaf. In the experiment, third or fourth leaf of the rice plant mounted in the chamber with a custom-made holder was used (Figure 6(C)). Precaution was taken to avoid damage to the leaf by having soft material such as cotton to cover the surface of the holder.

On illuminating two points on the leaf, two independent speckle fields were generated that interfere to make a random interference speckle pattern as shown in Figure 6(A). The speckle patterns were acquired by a CCD camera (XC-75, Sony Corp., Japan) set at a distance of 83 mm from the leaf. An interference filter (IF) was attached in front of the CCD camera to suppress the background light and select the probing light. The IF worked as a band-pass filter having a central wavelength of 633 nm with a bandwidth of 10 nm. A polarization filter (PF) was also used to maximize the interference signal. The distance *d* between the points of illumination on the leaf was set to be 3 mm. *d* can be adjusted by moving the prism P2 (Suruga Seiki, Japan) along the optical axis. P2 was mounted on a piezoelectric transducer (PZT) stage (E-620, Physik Instrumente, Germany) was used to give a certain phase shift between the interfering light beams. A frame grabber (VEC-PRO, IMPERX, USA) was used to acquire and store the interference speckle patterns every 500 msec.

Further, the probing area was covered with wheat flour to avoid light penetrating inside the leaf. Such penetration of light would result in generation of dynamic speckles from moving organelles within the leaf degrading the measurement accuracy of SIT. Such a dynamic speckle pattern is known as biospeckle (Aizu and Asakura 1996). Three halogen lamps with fiber optic light guides (PHL-150, Mejiro Precision Ltd., Japan) were used to provide the light intensity of 500 μmolm^-2^ sec^-1^.

SIT developed in our lab. is now available as a commercial product from Toyo Seiki Seisaku-sho, Ltd., Japan.

### Principle of SIT

SIT is a non-contact optical interferometric technique for measuring displacements at an accuracy of subnanometer and a temporal resolution of second (Kadono et al. 2001; Kadono and Toyooka 1991). Principle of statistical interferometry is different from that of conventional interferometric techniques. In a conventional interferometer, the light reflected from an optically flat reference mirror and reflected light from the sample under observation interfere to give an interference pattern. The phase change is derived from the interference pattern. It corresponds to the change of the optical path such as a displacement of the sample. In order to achieve subnanometer accuracy, stringent conditions were imposed on the interferometer that all of the uncertain factors in the interferometer, e.g. distortion of the reference mirror, vibration of the optical components, air turbulence, electric noise of the circuit and so on, has to be eliminated. Especially on the reference arm, it is required to make the resulting reference wavefront with no uncertainty such as deformity of reference mirror.

In contrast, in statistical interferometer, a completely random wavefront called a fully developed speckle field is used as reference and is generated when an optically rough surface is illuminated by a laser light. Phase of the speckle field has a uniform probability density function (PDF) that takes a constant probability density of 1/2π over the range from - π to π Characteristics of such speckle phase is remarkably stable and reliable and can play the role of a standard. To understand the principle of the method, one has to know the fundamental properties of the speckle field and its statistics that are given in (Additional file 1).

For every 500 milliseconds, the speckle interference patterns were acquired by a CCD camera and stored in a frame memory. Basically, when there is an in-plane elongation of *Δx* between the two illuminating points of the object, the optical path difference *ΔL* between the two interfering speckle fields can be expressed by,1

where *θ* is the angle between the illumination and the observation directions. Due to the elongation *Δx*, the phase difference between the two interfering light fields changes by *Ψ* and is given in terms of Δ*L* as:2

### SIT data analysis to obtain elongation rate

In our SIT algorithm, for deriving the unknown object phase *Ψ*, the statistical property of the speckle phase, i.e., the total randomness of the speckle phase was used as a constraint. A detailed basic SIT algorithm, and its extension developed by the authors, and derivation of the unknown object phase are given in (Additional file 1). The whole processing could be performed to get the true object phase with very high accuracy without any time consuming iterative procedure.

From the calculated phase change, corresponding elongation was calculated in real time. From the derived elongation over a period *τ* of 5.5 sec, the slope of the elongation data was obtained by linear regression. Relative elongation rate (RER) (nm mm^-1^ sec^-1^) is defined by,3

The following procedure was applied to obtain the RER and its fluctuations under control and O_3_ exposures: (a) Linear regression was done over 5.5 sec to obtain slope or to obtain instantaneous RER; (b) Repetition of procedure (a) for every 5.5 sec across the whole session of 7 hours; (c) The calculated RER was passed through a moving average filter with window size 165 sec to obtain smoothened data; (d) The smoothened data was subtracted out from the RER data of step (b) to eliminate a long-term trend. All calculations were done with Matlab (R2011b, MathWorks, USA). Statistical analyses were performed with the SPSS (Ver.19) statistical package. To analyze the statistical significance of the standard deviations obtained under different concentrations of O_3_, *t*-test was used.

### Plant cultivation and O_3_ exposure

**Figure 7:**
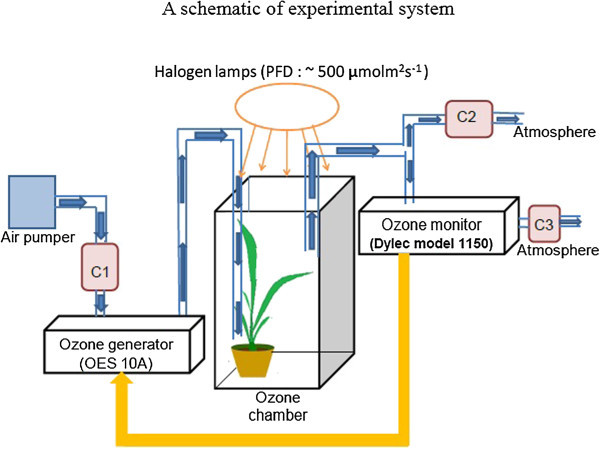
**A schematic of experimental system.** A schematic of experimental system for introducing Charcoal filtered air (CF air) and O_3_. Signal from the O_3_ monitor was fed back to the O_3_ generator to control precisely the concentration of O_3_ inside the chamber. Charcoal filter C1 was used to remove the ambient O_3_ in the environment. C2 and C3 were used to remove all O_3_ before releasing the air into the room. Flow rate was 10 l/min. Three halogen lamps with fiber optic light guides were used to provide light intensities of 500 μmolm^-2^ s^-1^ from the top of O_3_ chamber (not shown explicitly).

This research was carried out from December 2010 to May 2011 in Saitama University, Japan. Thirty to forty days old Koshihikari rice cultivar was used. We chose the age based on earlier studies (Inada et al. 2008; Yamaguchi et al. 2008). That study showed that during the vegetative period of thirty to forty days, exposing the cultivar to O_3_ had significant reduction on the relative growth rate, net assimilation rate, and photosynthetic rate. The culturing conditions were as follow:

1. The seeds of Koshihikari rice plant were pre-germinated in dark environment for four days.
2. Seedlings were transferred to separate plastic containers (600 ml volume cups) with two plants per container. Plastic containers were filled with the same soil sample with the required fertilizer, up to 300 ml of the cup.
3. The seedlings were placed in a growth chamber (Conviron, Controlled Environmental Ltd, Winnipeg, Manitoba, Canada.) for around three weeks. The conditions of the chamber were made to follow a day/night cycle of:
  - 12 hours/12 hours;
  - Light intensities of 260–350 μmol m^-2^ sec^-1^/0 μmol m^-2^ sec^-1^;
  - Temperatures were 25°C/18°C;
  - Relative humidity was kept at 55%–65% throughout a day;

Figure 7 shows a schematic of O_3_ exposure system consisting of O_3_ chamber (33.5 × 33.5 × 58 cm^3^) made up of glass and metal, an air pump, charcoal filters, an O_3_ generator (OES 10A, Dylec Inc.), and an O_3_ monitor (Model 1150, Dylec Inc.). The air from the air pump was passed through a charcoal filter (CF) to remove the ambient O_3_. The same inlet and outlet in the chamber were used for O_3_ and the charcoal filtered air. O_3_ concentrations were monitored with the O_3_ monitor, and the signal was fed back to the O_3_ generator to control O_3_ concentration precisely. The O_3_ feedback control system had an accuracy of around 0.5% during the experiment.

In O_3_ exposure measurements, the O_3_ generation system approximately took 10 minutes and 14 minutes to reach the set O_3_ concentrations, 120 nl l^-1^ and 240 nl l^-1^, respectively. Similarly the control system took approximately 10 minutes and 14 minutes to bring O_3_ concentration to 0 nl l^-1^ from 120 nl l^-1^ and 240 nl l^-1^, respectively. O_3_ concentrations used here were chosen based on the common criteria used in Japan. The condition of 120 nl l^-1^ corresponds to issuing warning while 240 nl l^-1^ corresponds to issuing a serious warning.

### O_3_ exposure timing protocol

At first, continuous CF air (10 liters/min.) was fed into the chamber for two hours. During this initial 2 hours, the stabilization of the whole system was carefully monitored, and then the data acquisition started. All the experiments were carried out at fixed times from 11.00 a.m. to 6.00 p.m. The entire experimental duration took 7 hours. The timing protocol used was as follows:

1. CF air was provided for the first 1 hour (control);
2. Next 3 hours O_3_ was introduced into the chamber;
3. For the last 3 hours, again CF air was used to fill the chamber.

Three conditions namely, the control with O_3_ concentration being 0 nl l^-1^, O_3_ exposure conditions of 120 nl l^-1^ and 240 nl l^-1^, were used. For each O_3_ exposure condition of control, 120 nl l^-1^, and 240 nl l^-1^, six samples were used. In control experiments, only CF air was provided during the entire duration of 7 hours.

Both control and O_3_ exposure experiments were conducted for three days consecutively. At the end of the each day, the plant used for experiment was kept in the growth chamber to be used on the following day. On second and third days, the same probing area of the leaf as of the first day (identified through the wheat flour on the leaf surface), was used to get the SIT measurements. The accumulated exposures over the threshold 40 nl l^-1^ (AOT 40) for three consecutive days were 240 nl l^-1^.h, 480 nl l^-1^.h, and 720 nl l^-1^.h for O_3_ exposure of 120 nl l^-1^, and 600 nl l^-1^.h, 1200 nl l^-1^.h, and 1800 nl l^-1^.h for O_3_ exposure of 240 nl l^-1^.

## Authors’ information

**BL Sanjaya Thilakarathne**

Reading for Ph,D in Environmental and Optical Sensing, Graduate School of Science and Engineering, Saitama University, Japan.

**Professor H. Kadono (Professor in Optics)**

Environmental Optical Sensing Laboratory, Graduate School of Science and Engineering, 255, Shimo- Okubo, Sakura-Ku, Saitama -shi, 338-8570, Japan.

**Uma Maheswari Rajagopalan Ph.D**

Research consultant, Integrative Neural Sytstems Lab, Brain Science Institute, Riken, 2-1 Hirosawa Wako-shi, Japan.

**Tetsushi Yonekura Ph.D**

Researcher, Center of Environment Science in Saitama, Kamitanadare 914, Kazo, Saitama, 347–0115, Japan.

## Electronic supplementary material

Additional file 1: **S1.** Fundamental statistical properties of the speckle field and the effect of non-spatial uniformity of object. **S2.** Algorithm of Statistical Interferometry. **S3.** Expansion of dynamic range in statistical interferometry. (DOC 2 MB)

## Abbreviations

SIT: Statistical interferometric technique
PDF: Probability density function
CCD: Charge coupled device
CF: Charcoal filtered
NIF: Nanometric intrinsic fluctuations
NNIF: Normalized nanometric intrinsic fluctuations
SD: Standard deviation
PZT: Piezoelectric transducer.
