# Supplementary material for: An optical interferometric technique for assessing ozone induced damage and recovery under cumulative exposures for a Japanese rice cultivar
Source: Springerplus. 2014 Feb 14;3:89. doi: 10.1186/2193-1801-3-89 (PMC3932158; doi:10.1186/2193-1801-3-89)
Supplement: Supplementary file 1 — Additional file 1: S1. Fundamental statistical properties of the speckle field and the effect of non-spatial uniformity of object. S2. Algorithm of Statistical Interferometry. S3. Expansion of dynamic range in statistical interferometry. (DOC 2 MB) [file 40064_2013_835_MOESM1_ESM.doc]

Supplementary information to the manuscript submitted to Springer plus

An optical interferometric technique for assessing ozone induced damage and recovery under cumulative exposures of for a Japanese rice cultivar

B.L.S. Thilakarathne, Uma Maheswari Rajagopalan, Hirofumi Kadono , Tetsushi Yonekura

|  |  |  |
| --- | --- | --- |
|  |  |  |
|  |  |  |

**S1. Fundamental statistical properties of the speckle field and the effect of non-spatial uniformity of object**

Speckles appear as a result of random interference of the scattered light by a rough surface object under the illumination of a coherent light such as a laser beam. The speckles were considered as a noise in the early advent of lasers. However, as theoretical interpretation of the laser speckle phenomenon developed, this phenomenon was widely recognized as an information source for the object under illumination. A large number of its applications were proposed and developed, e.g., surface roughness measurements, speckle velocimetry, stellar speckles, speckle interferometry, etc. The technique has also been applied for biological objects, e.g., blood flow measurements. These noninvasive measurement techniques using speckles have made them important making them no longer a random noise. Due to the randomness, it is important that a brief description on the statistical properties of the speckles (Dainty, 1984) becomes totally necessary for an interested reader to understand the basics of the technique used here in SIT (statistical interferometric technique).

In general, a speckle field is described as a complex optical field with an amplitude and a phase. In SIT, the statistical property of a fully developed Gaussian speckle field, i. e., a uniform probability density function of the speckle phase is utilized. Such a speckle field also shows related properties that the probability density function of the speckle intensity takes a negative exponential function resulting in the speckle contrast unity, C=1. In order to generate such a fully developed speckle field, only two conditions are needed to be satisfied. One is related to the optical roughness *σ_o_* of the object and the other is related to the correlation length *ξ* of the surface height variations. The optical roughness *σ_o_*, standard deviation of optical path variation due to surface height variation of the object, must be larger than the wavelength of the light used in the system. In our case, as a reflection setup was used, the surface roughness *σ_h_*, standard deviation of the surface height variation of the object, must be larger than half of the wavelength of the light used. Therefore in the optical setup shown in Fig.S1, the main condition that needs to be fulfilled is that the roughness is,


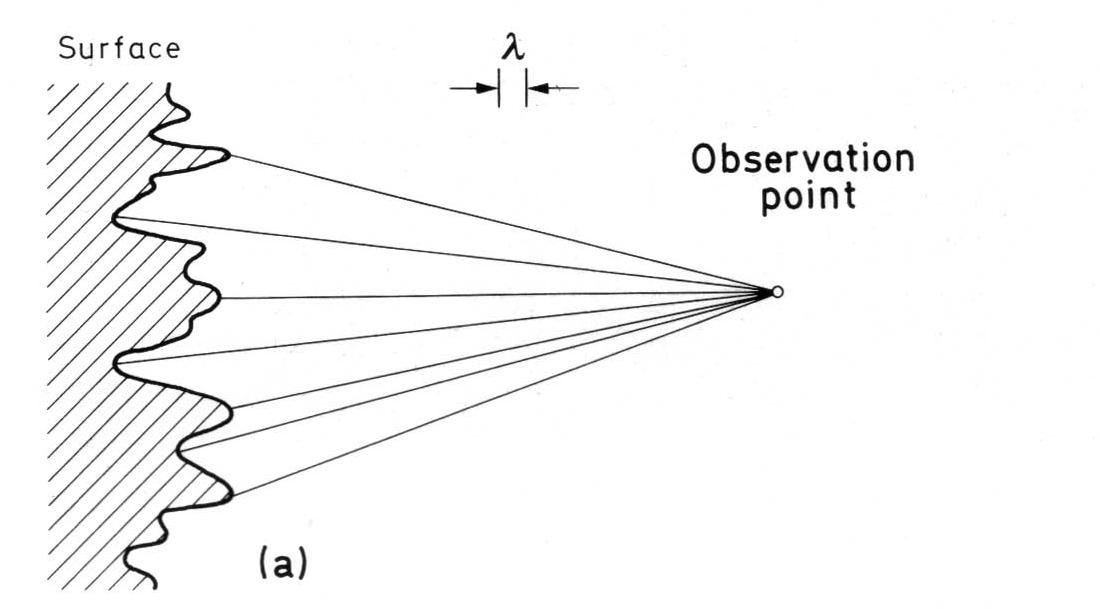


Fig.A1 S1 Complex speckle amplitude in diffraction field.

Observation point

Surface

$\sigma_{o}>>\lambda or \sigma_{h}>>\lambda/2$ (S1)

With respect to the correlation length that quantifies surface height variations, the number of scatterers *N* based on the discrete scatterer model is commonly introduced. *N* is the number of the scatterers contributing to the formation of the speckle field and is defined by

$N=\frac{\text{area of illumination} }{\text{area of elementary scatterer}}=\frac{\pi w^{2}}{\pi\xi^{2}},$ (S2)

where *w* is the radius of the probing beam, and *ξ* is the correlation length of the surface height variations.

Our method is based on the statistics of Gaussian speckle field, the discussion has been restricted to Gaussian speckle field. In other words this means the complex speckle amplitude obeys Gaussian statistics according to the central limit theorem. In practice, this requirement is well satisfied with the condition,

$N>10.$ (S3)

Here, let us define the j-th elementary wave from j-th scatterer contributing to the formation of the speckle field at a certain observation point P be *a_j_*. The complex speckle amplitude *A* at the observation point P in a diffraction field (Fig.S1) is given by,

$A=A_{r}+iA_{i}=\sum_{j=1}^{N} a_{j}=\sum_{j=1}^{N} \left|a_{j}\right|\exp\left(i\varphi_{i}\right).$ (S4)

Here, suffixes r and i denote real and imaginary parts of the complex number, respectively. When both of the conditions given by Eqs.(S1) and (S3) are satisfied, the statistics of the complex speckle amplitude becomes zero-mean circular Gaussian statistics. This means,

$\begin{matrix} <A>=<A_{r}>+i<A_{i}>=0, \\ \sigma_{r}=\sigma_{i}, \\ \rho=<A_{r}A_{i}>/\sqrt{\sigma_{r}\sigma_{i}}=0. \end{matrix}$ (S5)

In Eq.(S5), <…> stands for ensemble average. *σ_r_* and  *σ_i_* are the standard deviations of the real and imaginary parts of the complex speckle amplitude of A, respectively. *ρ* is the correlation coefficient of the real and imaginary parts of the complex speckle amplitude. In general, first order statistics of the Gaussian speckle field is described perfectly by means of the joint probability density function *p_r,i_(A_r_, A_i_)* of real and imaginary parts of the complex speckle amplitude, and takes two dimensional Gaussian distribution on the complex amplitude plane of *A_r_* and *A_i_* as shown in Fig.S2. When the conditions of Eqs.(S1) and (S3) are satisfied, one can see that the contour line of a certain value of the probability density, i.e., equi-probability density ellipse, becomes circular.

When the optical roughness is smaller and does not satisfy the condition of Eq.(S1), so called partially developed speckle field is produced. Although the statistics of the complex speckle field sill obeys Gaussian statistics with the condition of Eq.(S3), the mean *<A>* and the correlation ρ take certain values, and *σ_r_* and  *σ_i_* are not equal in general. This implies that the equi-probability density ellipse deviates from the origin and becomes elliptic. The statistics of such a speckle field is called non-circular Gaussian statistics. The significant point of the partially developed speckle field is that its statistical properties are dependent on the surface roughness and the number of scatterers *N*. In other words, the speckle contrast and the probability density distribution of the speckle phase are dependent on those two parameters in very complex manner .

However, the roughness becomes larger and satisfies the condition of Eq.(S1), the dependence of the speckle field on N becomes weaker and weaker. Finally when the condition of the roughness given by Eq.(S1) is satisfied, the statistics of the speckle field does not depend on these parameters, *σ_o_* and *N*. Therefore the fully developed speckle field is a very stable and a kind of saturated field.

In addition, the discussion above is the case that illumination and the observation directions are parallel and normal to the object surface where a specular reflection from the object is superposed onto the scattered component. As in the optical system used in our experiment, we observed the speckles in off axial diffraction field. In this case, as the observation direction deviates from the normal to the object surface, the speckle field quickly approaches to the fully developed speckle field even if the optical roughness of the object doesn’t satisfy the condition of Eq.(S1). A detailed theoretical analysis of the statistical properties of the off axis diffraction speckle field is given in our previous study . Although the wheat flour was not homogenized, due to the large optical roughness of the wheat layer together with the off-axis observation, the statistics of the field where SIT is based on was never affected by the spatial non-uniformity of the grain of wheat flour in practice. This was well coincide with the experience in our experiment.

**S2. Algorithm of Statistical Interferometry**

In the optical system shown Fig.1A, the random speckle interference patterns corresponding to the elongation, *Δx*, of the object are stored in a frame memory through the CCD camera as *I(x,t_1_)*,*I(x,t_2_)*,･･･,*I(x,t_n_),* at times *t* = *t_1_*,*t_2_*･･･*t_n_*. Phasors corresponding to the phase change due to the elongation of the object are shown in Fig.S3.

Next, we consider three images, *I_1_(****x****), I_2_(****x****), I_3_(****x****)*, that are arbitrarily chosen from the frame memory, respectively, at times *t=h_1_, h_2_*, and *h_3_*. The intensity distributions of those three random interference patterns are expressed by,

$\begin{matrix} I_{1}\left(x\right)=I_{0}\left(x\right) \left[1+\gamma\left(x\right)\cos\left(\varphi\left(x\right)+\psi_{1}\right)\right] _{,} \\ I_{2}\left(x\right)=I_{0}\left(x\right) \left[1+\gamma\left(x\right)\cos\left(\varphi\left(x\right)\right)\right] _{,} \\ I_{3}\left(x\right)=I_{0}\left(x\right) \left[1+\gamma\left(x\right)\cos\left(\varphi\left(x\right)+\psi_{3}\right)\right] \end{matrix}$, (S6)

where$I_{0}\left(x\right)$ and $\gamma\left(x\right)$ are, respectively, the average intensity and the modulation factor at each point ***x*** on the observation plane.*ψ_1_* and *ψ_3_* are object phase difference between *t=h_1_* and *h_2_*, and *t=h_2_* and *h_3_,* respectively*,* and those values are to be derived.

Here, we briefly describe the way to derive the object phase (Kadono *et al.* 2001). Since three systems of equations, Eq. (S6), have five unknown variables, *I_o_, γ, ψ_1_, ψ_3_*, and *φ,* we cannot calculate *ψ_1_* and *ψ_3_* directly. In the next step, we assume a certain value for the phases *ψ_1_* and *ψ_3_* as,

$-\psi_{1}=\psi_{3}=\psi_{v}.$(S7)

The phase term *ψ_ν_* is referred to as a virtual phase whose value can be chosen arbitrarily. The phase of the speckle field is derived from Eq.(S6) with the assumption of Eq. (S7) as,

$\varphi^{'}\left(x\right) = \tan^{-1}\frac{I_{1}\left(x\right) -I_{3}\left(x\right)}{I_{1}\left(x\right) + I_{3}\left(x\right) -2I_{2}\left(x\right)}\cdot\frac{\cos\psi_{v}-1}{\sin\psi_{v}}.$ (S8)

*φ’(****x****)* is referred to as an evaluated phase of the speckle field. The assumption given by Eq.(S7) is not reasonable in general. As * φ’(****x****)* differs from the true phase* φ(****x****)*of speckle field, the PDF of the evaluated phase* φ’(****x****)* of the speckle field doesn’t take the uniform distribution of 1/2π. This implies that, by making a proper correction for the assumption of Eq.(S7) based on the deviation of the PDF of the evaluated phase of speckle field from the uniform distribution, the actual object phase can be derived. The deviation from the uniform distribution becomes prominent even for a fairly small phase difference from the virtual phase value *ψ_v_* for the object phase. To explain more precisely, two phase components, symmetrical *Δψ*_s_ and antisymmetrical phase deviations *Δψ*_a_, are introduced. Using these variables the object phase can be expressed by,

$\begin{matrix} \psi_{1}=-\psi_{v}-\Delta\psi_{s}+\Delta\psi_{a}, \\ \psi_{3}=\psi_{v}+\Delta\psi_{s}+\Delta\psi_{a}. \end{matrix}$(S9)

According to the precise analysis, the symmetrical and the antisymmetrical deviations from the virtual phase cause nonuniform deviations expressed with cos2*φ'* and sin2*φ'* into the PDF of the evaluated phase of speckle field, respectively. These two types of deviation from the uniform PDF gives us a way to determine two phase components, *Δψ_s_* and *Δψ_a_*,. Thus the object phases, *ψ_1_* and *ψ_3_*, can be uniquely determined. Through changing the combination of three frames and repeating the same procedure, whole phase change of the object can be obtained.

The advantage of the method is that, because of the statistical basis of the method, improvement of the accuracy is inherently assured by taking more data samples into account, and the accuracy of λ/1000 can be easily achieved with approximately 40000 samples of data according to the computer simulation. However, the elongation of the object that can be determined has been limited to phase range less than 2π.

**Dainty JC**. 1984. *Laser Speckle and Related Phenomena (Topics in Applied Physics)*. Berlin: Springer-Verlag.

**Kadono H, Asakura T**. 1985. Statistical properties of the speckle phase in the optical imaging system. *Journal of Optical Society of America A* **2**, 1787-1792.

**Kadono H, Takai, N.and Asakura, T.** 1986. Statistical properties of the speckle phase in the diffraction region. *Journal of Optical Society of America A* **3**, 1080-1089.

**S3. Expansion of dynamic range in statistical interferometry**

First we describe the practical procedure to acquire the interference patterns for obtaining the object phase as described in the previous section, and then the dynamic range of the measurement is expanded by improving the algorithm (Kobayashi and Kadono, 2010).

The reference interference patterns, *I_1_* and *I_3_* , are acquired by introducing appropriate phase modulations $-\psi_{r}$ and $+\psi_{r}$ between the two illuminating beams through the use of a phase modulator (PZT). The phase modulation of ±*ψ_r_* are introduced only at the beginning to assure the phase difference between three frames in combination because the combination of the very close phasors increase the error. Those phase modulations are shown in Fig.S4 A by using phasors.

The interference patterns due to the object elongation are recorded continuously in a frame memory. The phase due to the object change at time *t_i_* of i-th frame is indicated by ∠Phasor_2,i_, and the interference speckle pattern at time *t_i_* is denoted by *I_2,i_*. Therefore, the phase difference between the reference patterns and the initial object phase are given by,

$$\begin{matrix} \angle\text{Phasor}_{1}=\angle\text{Phasor}_{\text{2,1}}-\psi_{r}, \\ \angle\text{Phasor}_{3}=\angle\text{Phasor}_{\text{2,1}}+\psi_{r}. \end{matrix}$$

(S10)

∠･･･ shows the angle of the phasor. The object phase is calculated from the combination of three frames of *I_2,i_* with *I_1_* and *I_3_* fixed. The object phase change $\Delta\psi\left(t_{i}\right)$ at *t_i_* is given with the explicit indication of the combination of three interference patterns as;

$\Delta\psi\left(t_{i}\right)=\left.\psi\left(t_{i}\right)\right|_{I_{1},I_{2, i},I_{3}}-\left.\psi\left(t_{1}\right)\right|_{I_{1},I_{2, 1},I_{3}}, \left(i=1,2,3\text{･･･}n\right).$ (S11)

Here, *n* is the number of the interference patterns acquired for the object. The object elongation is given by, $\Delta x\left(t_{i}\right) = \Delta\psi(t_{i})\lambda/2\pi\sin\theta$.

If the Phasor_2,i_ is not close to the reference Phasor_1_ or Phasor_3_, the object phase can be obtained with a fairly small error. As the Phasor_2,i_ approaches very close to the reference Phasor_1_ or Phasor_3_, the error in the calculated object phase would become larger because the difference in the interference pattern becomes smaller. In addition, the object phase error also increases when the decorrelation between *I_2,i_* , *I_1_* ,and *I_3_* occurs due to either translation of object or change of surface structure.

$\begin{matrix} \angle\text{Phasor}_{1}' =\angle\text{Phasor}_{\text{2,k}}-\psi_{r}, \\ \angle\text{Phasor}_{3}' =\angle\text{Phasor}_{\text{2,k}}+\psi_{r}. \end{matrix}$Therefore, when the object phase $\Delta\psi\left(t_{k}\right)$exceeds a certain threshold $\psi_{th}$, i.e. $\Delta\psi\left(t_{k}\right) \geq\psi_{th}$, the reference patterns of Phasor_1_ or Phasor_3_ have to be renewed as *I_1_’* and *I_3_’* using the phase modulator with the modulation $\pm\psi_{r}$ for Phasor_2,k_ (Fig.S4B). The renewed phasors satisfy the following relations:

(S12)

Therefore, the object phase at *t_j_*, after the renewal of the reference pattern is given by,

$\Delta\psi'\left(t_{j}\right) = \left.\psi\left(t_{k+j}\right)\right|_{I'_{1},I_{2, (k+j)},I'_{3}} - \left.\psi\left(t_{k}\right)\right|_{I'_{1},I_{2, k},I'_{3}}, \left(j=1,2,3\text{･･･}n-k\right).$ (S13)

Then, corresponding elongation after the renewal is given by,

$$\Delta x'\left(t_{j}\right) = \Delta\psi'(t_{j})\lambda/2\pi\sin\theta.$$

(S14)

The total object elongation at time *t_i_*, ( *i*=*k+j* ), is expressed by,

　 $\Delta x\left(t_{i}\right) = \Delta x\left(t_{k}\right)+\Delta x'\left(t_{j}\right) .$ (S15)

The renewal of the reference patterns are also carried out when the number of invalid phase data exceeds a certain threshold, and the modulation factor$\gamma\left(x\right)$ does not satisfy a given threshold. One of the reason for the invalid phase data is the decorrelation of the speckle patterns.

When the condition $\left|\Delta\psi'\left(t_{j}\right) \right| <\psi_{th}$does not hold again, the reference patterns are renewed. In practice, the threshold *ψ_th_* was set to be fairly smaller value of 2π/10 that corresponds to the elongation of the object 0.17μm with the observation angle *θ*=22 degree. This resulted in the frequent renewal of the reference speckle patterns. By repeating the renewal of the reference patterns, the algorithm made it possible to measure the phase changes of the object that were much larger than 2$\pi$ with robustness to speckle decorrelation and unexpected external turbulence. In addition, the data processing unit of the measurement software was designed so as to continuously monitor the invalid data points in the acquired images and report the number of invalid data points which were mainly due to the decorrelation of speckle patterns and electrical noise. In this way, we could monitor and assure the quality of measurements and data acquisition.

**Kobayashi K, Kadono H**. 2010. Expansion of the dynamic range of statistical interferometry and its application to extremely short- to long-term plant growth monitoring. *Appl. Opt.* **49**, 6333-6339.


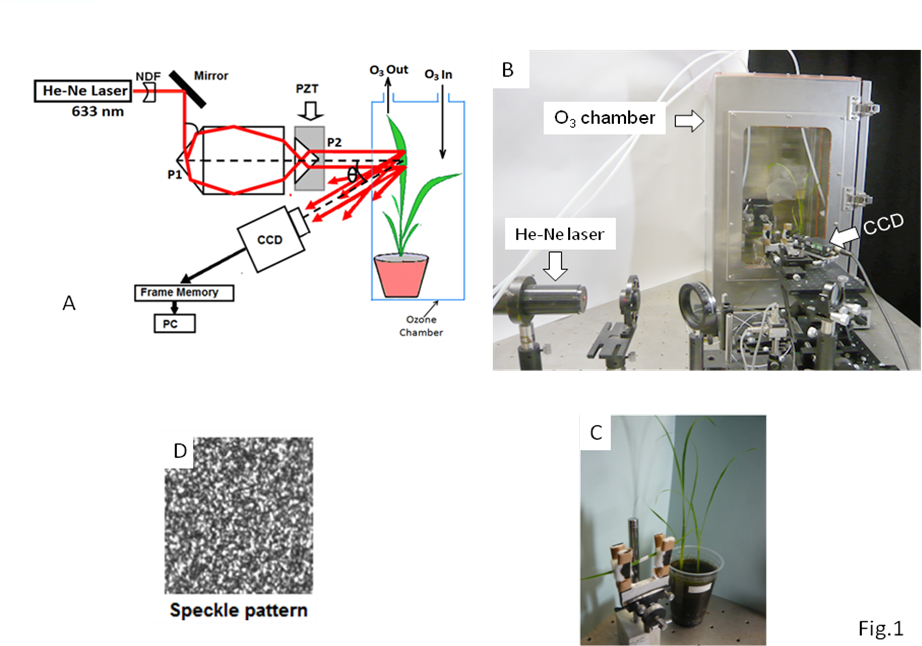
***
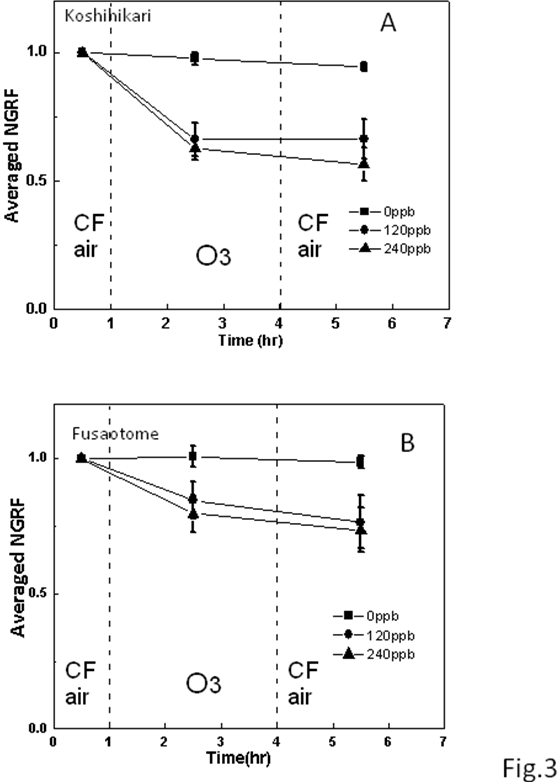
*
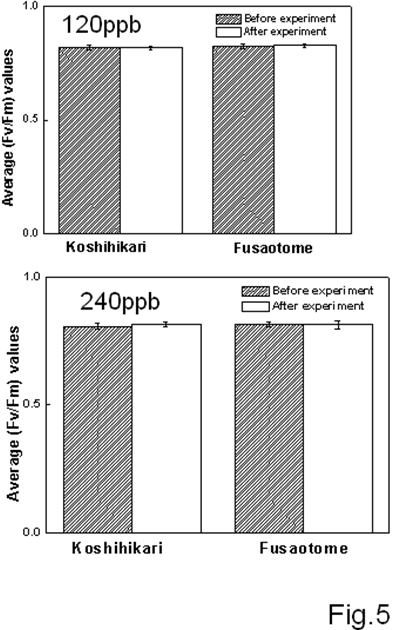

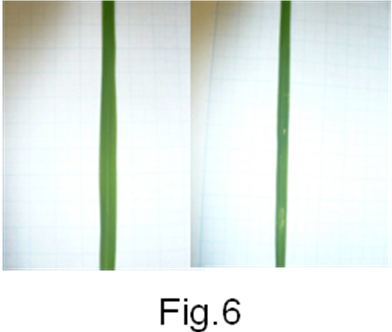
*Captions for Figures***

Figure S1 Complex speckle amplitude in diffraction field.

Figure S2 Joint probability density distribution of complex speckle amplitude.

Figure S3 Phasors corresponding to the phase changes that are due to the elongation of the object. (Under request for copy right permission from OSA)

Figure S4 Expansion of the dynamic range,(A) before, and (B) after renewal of reference patterns, Phasor_1_ and Phasor_3_. (Under request for copy right permission from OSA)

Fig.A2 S2 Joint probability density distribution of complex speckle amplitude

Fig.S1 Complex speckle amplitude in diffraction field.

**
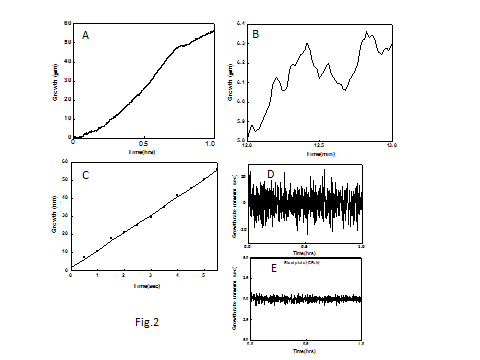
**
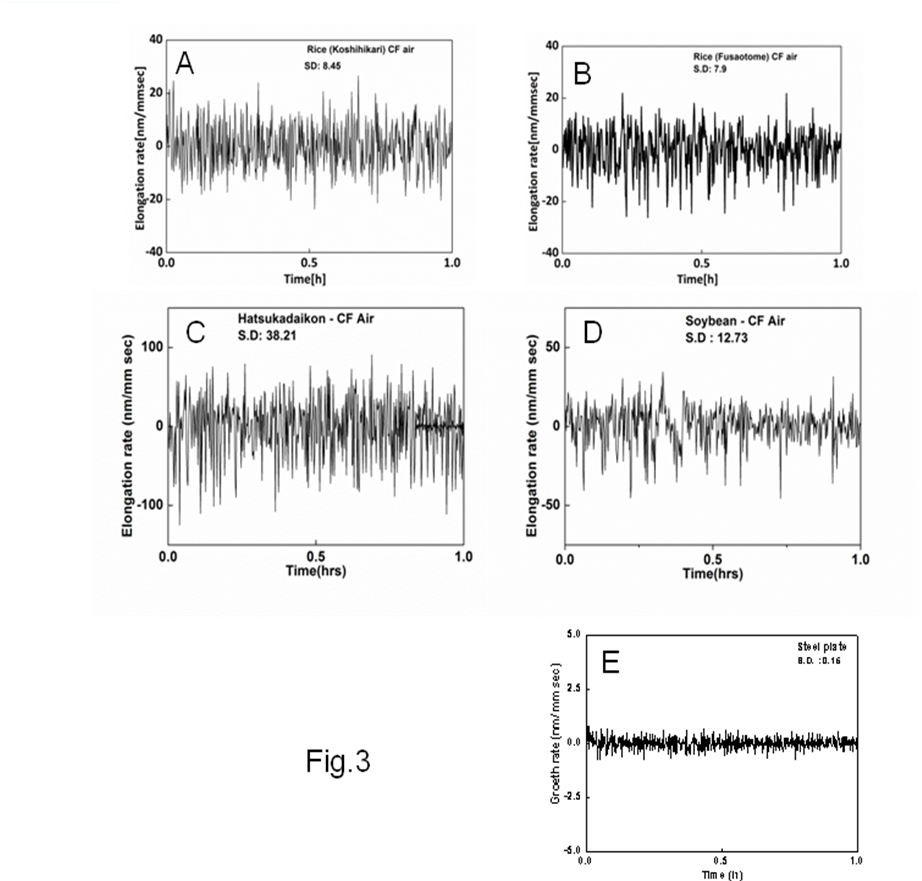
 28, 1021-1036.

Fig.S3 Phasors corresponding to the phase changes that are due to the elongation of the object

Fig. S7 NIF of Fusaotome under 240ppb ozone exposure with probing beam of 532nm

28, 1021-1036.

28, 1021-1036.
